# Factors influencing p53 expression in ovarian cancer as a biomarker of clinical outcome in multicentre studies

**DOI:** 10.1038/sj.bjc.6603300

**Published:** 2006-08-01

**Authors:** P de Graeff, J Hall, A P G Crijns, G H de Bock, J Paul, K A Oien, K A ten Hoor, S de Jong, H Hollema, J M S Bartlett, R Brown, A G J van der Zee

**Affiliations:** 1 Department of Gynaecologic Oncology, University Medical Centre Groningen, University of Groningen, Groningen 9700 RB, The Netherlands; 2 Centre for Oncology and Applied Pharmacology, Cancer Research UK Beatson Laboratories, University of Glasgow, Garscube Estate, Glasgow G61 1BD, UK; 3 Department of Epidemiology and Statistics, University Medical Centre Groningen, University of Groningen, Groningen 9700 RB, The Netherlands; 4 Department of Medical Oncology, University Medical Centre Groningen; University of Groningen, Groningen 9700 RB, The Netherlands; 5 Department of Pathology, University Medical Centre Groningen, University of Groningen, Groningen 9700 RB, The Netherlands; 6 Endocrine Cancer Group, University Department of Surgery, Royal Infirmary, Glasgow G31 2ER, UK

**Keywords:** ovarian cancer, prognosis, p53

## Abstract

The prognostic impact of p53 immunostaining in a large series of tumours from epithelial ovarian cancer patients in a two-centre study was analysed. The study population (*n*=476) comprised of a retrospective series of 188 patients (Dutch cohort) and a prospective series of 288 patients (Scottish cohort) enrolled in clinical trials. P53 expression was determined by immunohistochemistry on tissue microarrays. Association with progression-free survival (PFS) and overall survival (OS) was analysed by univariate and multivariate Cox regression analysis. Aberrant p53 overexpression was significantly associated with PFS in the Dutch and Scottish cohorts (*P*=0.001 and 0.038, respectively), but not with OS in univariate analysis. In multivariate analysis, when the two groups were combined and account taken of clinical factors and country of origin of the cohort, p53 expression was not an independent prognostic predictor of PFS or OS. In this well-powered study with minimal methodological variability, p53 immunostaining is not an independent prognostic marker of clinical outcome in epithelial ovarian cancer. The data demonstrate the importance of methodological standardisation, particularly defining patient characteristics and survival end-point data, if biomarker data from multicentre studies are to be combined.

Ovarian cancer is the leading cause of death from gynaecological cancer in the Western world. Overall survival (OS) for patients with advanced disease (stage III and IV according to the International Federation of Gynaecology and Obstetrics (FIGO); Cancer Committee of the International Federation of Gynaecology and Obstetrics, 1986) is only 15–25% at 5 years (Kristensen and Trope, 1997). Clinical decision-making is currently based on so-called ‘classical’ clinicopathological prognostic factors such as tumour stage, differentiation grade and histomorphologic tumour type. However, these prognostic factors do not allow viable prediction of the outcome for the individual patient. Biological behaviour of the tumour, response to chemotherapy and overall patient survival vary greatly between apparently similar cases (Friedlander, 1998). Identification of new prognostic factors would be of great importance in predicting disease outcome, and therefore guiding therapeutic choices (Arts *et al*, 2000).

One of the most studied prognostic markers in ovarian cancer so far is the tumour suppressor gene *p53*. The p53 protein plays a key role in cell cycle regulation and suppression of tumour development. DNA damage results in increased levels of p53, which lead to cell cycle arrest in G1 phase, followed by DNA repair or apoptosis (Levine, 1997; Vogelstein *et al*, 2000). Mutations of the *p53* gene as determined by mutation analysis and/or positive immunohistochemical (IHC) staining for p53 are common in ovarian cancer and have been associated with poor clinical outcome. However, results of the many studies on the prognostic value of p53 expression in ovarian cancer are inconclusive (Marks *et al*, 1991; Hartmann *et al*, 1994; Van Der Zee *et al*, 1995; Allan *et al*, 1996; Anttila *et al*, 1999; Ferrandina *et al*, 1999; Reles *et al*, 2001; Nakayama *et al*, 2003; Nielsen *et al*, 2004). One of the most important reasons for these conflicting results is the considerable methodological variability among the different studies (Hall *et al*, 2004). The type of study design, assays used to study p53 expression, determination of cutoff points for aberrant p53 expression and the definition of study end points vary greatly among different studies. Furthermore, most studies have a small sample size and include patients with different treatment regimens (Hall *et al*, 2004).

The aim of the present study was to investigate the prognostic and predictive value of p53 expression in tumour samples from a large group of ovarian cancer patient with clinical data collected through centres in the United Kingdom and the Netherlands, and to test the hypothesis that p53 status could be a reproducible marker for clinical outcome following therapy in ovarian cancer. We aimed to minimise variability in the study by using well-defined patient populations, and by performing tissue microarray (TMA) construction, IHC staining and scoring at one location.

## PATIENTS AND METHODS

### Study design and inclusion criteria

**Figure 1:**
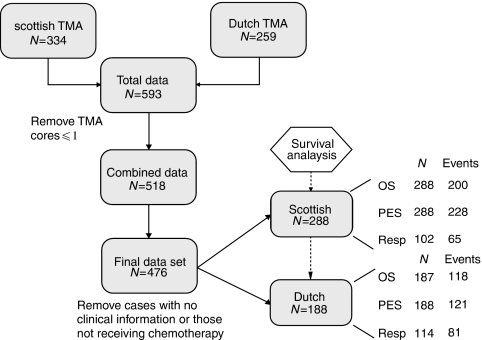
A diagram illustrating the flow of patients through the study. p53 staining in ovarian cancer tissue samples was analysed by TMA and IHC. Data sets (blue boxes) from the Netherlands and Scotland were combined. Analyses (white hexagons) and reasons for patient drop out are indicated.

Our study population comprised of retrospective (188 Dutch patients) and prospective (288 Scottish patients) data. Figure 1 describes the flow of patients through the study. In both the Dutch and Scottish cohorts, the principal eligibility criterion was primary chemonaive epithelial ovarian cancer of any histological subtype or stage. Patients were excluded if they had benign and borderline tumours, if they did not receive chemotherapy or if no clinical and follow-up information was available. Furthermore, all cases with <2 evaluable cores on TMA were excluded from analysis. Wherever possible, we aimed to comply with the recently published REMARK criteria for the reporting of prognostic factor studies (McShane *et al*, 2005).

### Patients, treatment and follow-up for Dutch patients

Since 1985, clinicopathologic and follow-up data of all malignant epithelial ovarian cancer patients treated at the Department of Gynaecological Oncology at the University Medical Centre Groningen have been prospectively stored in a computerised database. We retrospectively analysed the data of all patients treated from 1985 to 2002 for which paraffin-embedded tumour tissue was available.

Primary treatment for all patients consisted of surgery. The standard surgical procedure was total abdominal hysterectomy, bilateral salpingo-oophorectomy, omentectomy, multiple peritoneal biopsies and peritoneal washings with cytology. All patients were staged according to the FIGO classification (Cancer Committee of the International Federation of Gynaecology and Obstetrics, 1986). Tumours were graded and classified by a gynaecological pathologist according to WHO criteria (Scully, 1999). Adjuvant chemotherapy consisted of different platinum-based treatment regimens. Response to chemotherapy was assessed using WHO criteria (World Health Organization, 1979). After chemotherapy, patients were followed up to 10 years with gradually increasing intervals. Follow-up data were completed for all patients until March 2005. Median follow-up of patients still alive at the time of analysis was 51.6 months (range 2.8–136.5 months).

### Patients, treatment and follow-up for Scottish patients

Data from eight previous multicentre, UK and international clinical trials managed through the Beatson Oncology centre, CRUK Trials office, Glasgow, since 1989–2003 were stored in a computerised database. Thirty-seven (12.8%) patients from the Scottish cohort were recruited from outside the UK. The median follow-up of patients still alive at the time of analysis was 44.3 months (range 1.32–137.4 months). Patients underwent surgery, followed by randomisation onto an arm of the trial. Patients were staged according to the FIGO classification, graded by WHO criteria and all patients received adjuvant chemotherapy consisting of platinum-based regimes, single-agent taxanes and other chemotherapy regimes including melphalan and etoposide. Response to chemotherapy was determined by either modified SWOG criteria or radiological findings (Vasey *et al*, 2004).

### Institutional review board approval

For Dutch patients, clinicopathological and follow-up data were obtained during standard treatment and follow-up. For the present study, all relevant data were retrieved from our database into a separate anonymous database. In this separate database, patient identity was protected by a study-specific, unique patient code, which was known to only two dedicated data managers, who also have responsibility for the larger database. In case of uncertainties with respect to clinicopathologic and follow-up data, the larger database could only be checked through the data managers, thereby ascertaining the protection of patients' identity. Owing to these precautions for this study, no further institutional review board approval was needed, according to Dutch law. For the Scottish data, ethical approval was obtained from the relevant MREC and LREC committees.

### Tissue microarray construction

Tissue microarrays were constructed as described in previous studies (Kononen *et al*, 1998; Hoos and Cordon-Cardo, 2001). In summary, paraffin-embedded tumour tissue blocks and matching haematoxylin–eosin (H&E)-stained slides were retrieved from the pathology archives and representative areas of tumour were marked on each H&E-stained slide. Four cores of 0.6 mm^2^ were taken from each donor block and arrayed on a recipient paraffin block using a precision instrument (Tissue Arrayer, Beecher Instruments, Silver Spring, MD, USA). Using a microtome, 5 *μ*m sections were cut from each TMA block and applied to aminopropyltriethoxysilane-treated slides. All sections were stained within 2 weeks of sectioning. The presence of tumour tissue on the arrayed samples was verified on an H&E-stained section.

For the Scottish group, donor blocks were retrieved from patients recruited into seven clinical trials and TMAs were constructed separately for each trial. For the Dutch group, tumour tissue was arranged in eight TMA blocks. Duplicate cores of five tumour samples, an ovarian cystadenoma and normal tissue (fallopian tube, endometrial, endocervical and cervical tissue) were included on each TMA block to ensure similarity of staining between the slides and to study p53 expression in normal tissues.

### Immunohistochemical staining of TMAs

Tissue microarray sections were dewaxed in xylene and rehydrated through graded concentrations of ethanol to distilled water. For antigen retrieval, the sections were boiled with ethylenediaminetetraaceticacid buffer (pH 8) in a microwaveable pressure cooker for 5 min at full power.

Staining was performed in a Dako Autostainer (Dako, Cambridgeshire, UK). Endogenous peroxidase activity was blocked by incubating the slides in Dako Peroxidase Block for 5 min. The sections were incubated with normal goat serum for 20 min, followed by incubation with the primary antibody for 30 min at room temperature. The monoclonal mouse anti-human antibody DO-7 (dilution 1 : 2000; Dako), which detects both wild-type and mutant p53 protein, was used as the primary antibody. Detection was by a goat anti-mouse/rabbit secondary antibody conjugated with a peroxidase-labelled polymer (Dako EnVision+ system). The antigen–antibody reaction was visualised with 3,3′-diaminobenzidine for 10 min and was enhanced in copper sulphate (5 min). Sections were counterstained with haematoxylin. Separate full slides containing breast cancer tissue of known p53 status were used as external positive and negative controls for p53 staining.

Two observers (PG and KH) independently scored IHC staining of all TMAs without prior knowledge of the clinicopathological information. The cases with a discrepant score by the two observers were re-examined with a gynaecological pathologist, until consensus was reached. Immunoreactivity for the DO-7 antibody was scored according to the intensity of nuclear staining and to the percentage of positively stained tumour cells. Tumours showing >50% immunostaining with moderate or strong intensity were considered as having aberrant p53 immunostaining. This cut point was based on the observation of weakly positive immunostaining in normal control tissues.

### Statistical design and study end points

Statistical analysis was performed using the SPSS 12.01 software package (SPSS Inc., Chicago, IL, USA). The three end points investigated were progression-free survival (PFS), OS and response to chemotherapy. Progression-free survival was defined as date of surgery (Dutch) or randomisation on the trial (Scottish: within 6 weeks of surgery) until progression or death. Overall survival was defined as date of surgery or randomisation onto the trial until death. Response to chemotherapy was assessed by CA125 measurement, modified SWOG or RECIST criteria (Scottish cohort) and WHO criteria (Dutch).

As ‘classic’ clinically useful clinicopathological factors, such as stage, distinguish risk groups with a hazard ratio (HR) of approximately 2, we set this as the target size of effect for p53. Standard calculations were used to assess the power of the analysis (Schmoor *et al*, 2000). The Dutch (*N*=188) and Scottish (*N*=288) studies individually had a power of 95.7 and 99.5% to detect a HR of 2, assuming a frequency of p53 abnormalities at 50 and 40% censoring. To detect the more subtle effect size of HR 1.5, the power of the respective studies was 57.7 and 76.0% (or 92.6% for combined data).

Differences between the two patient groups were analysed using *χ*^2^ tests for clinicopathological characteristics, and Kaplan–Meier estimates for PFS and OS. *χ*^2^ tests were used to assess associations between p53 expression and clinicopathological characteristics or response to chemotherapy. Survival analysis was performed using Cox proportional hazards model. The cut point for aberrant p53 staining was decided *a priori*, as described above, and p53 was entered as a categorical variable. Categorised variables used for univariate analysis included age (<58 or ⩾58 years), stage (stage I/II or stage III/IV), grade (grade I or grade II/III), histology (serous or non-serous), residual disease (<2 or ⩾2 cm) and type of chemotherapy (platinum, platinum/taxane or other). Univariate analysis was stratified for chemotherapy. All variables, including country of origin, were subsequently included in multivariate analysis. For multivariate analysis of response to chemotherapy, logistic regression was used. For this analysis, response was entered as a categorical variable (complete and partial response *vs* stable and progressive disease). To investigate if the country of origin of the data or the type of chemotherapy affected the relationship of p53 with clinical outcome, interaction tests were performed within a Cox regression model. The 5% confidence level was used to test for significance of interactions. All *P*-values were two sided.

## RESULTS

### Clinicopathologic characteristics

Clinicopathologic data from both patient populations, separately and combined (*N*=476), are summarised in Table 1. Adjuvant chemotherapy consisted of a platinum-containing regimen in 195 (41.0%) patients and a platinum- and taxane-containing regimen in 237 (49.8%) patients. Fourty-four (9.2%) patients were treated with other treatment regimens, including melphalan and etoposide. Median PFS for the whole cohort was 14.7 months (95% confidence interval (CI): 12.8–16.5) and median OS was 30.6 months (95% CI: 25.6–35.7).

Analysis of differences between the two patient groups showed that the Scottish cohort had a higher proportion of cases with smaller residual disease (49.6 *vs* 38.3%; *P*=0.020), higher grade tumours (92.4 *vs* 83.8%; *P*=0.006) and proportion of patients receiving platinum/taxane combination therapy (57.3 *vs* 38.3%; *P*=0.0002). All other factors were not significantly different between the two data sets (age, *P*=0.99; stage, *P*=0.82 and histology, *P*=0.71). The Scottish cohort had worse PFS than the Dutch (*P*=0.023). The same trend was observed for OS, but this effect was not significant (*P*=0.073).

### Immunohistochemistry

Frequencies of p53 staining intensity and percentage of positively stained cells were equally distributed across the Dutch and Scottish group (Table 1). The intensity of p53 staining was normal in 228 (47.9%) samples, and elevated in 248 (52.1%) samples.

### Prognostic and predictive value of aberrant p53 staining, scored for the Dutch and Scottish group separately

Owing to differences in the clinical characteristics of the cohorts, we firstly performed our analysis for the Dutch and Scottish group separately. Table 2 shows the relationship between p53 staining and clinicopathological characteristics for the two patient groups separately. For UK patients, excessive p53 staining was associated with a high differentiation grade (*P*=0.003), but not with other adverse prognostic factors, such as a higher age, late stage disease, a serous tumour type and >2 cm residual disease. In the Dutch group, a correlation existed between excessive p53 staining and late-stage disease (*P*=0.006), a serous tumour type (*P*=0.04), a high differentiation grade (*P*<0.001) and >2 cm residual disease (*P*=0.002). Again, there was a lack of association between excessive p53 staining and higher age. Investigating the apparent difference in the relationship between p53 and clinical factors in the two cohorts, a multivariate logistic regression suggested that only grade was a significant predictor of p53 status (*P*=2.07e-5, odds ratio (OR)=8.45, CI: 3.16–22.6) whereas all other factors, including patient cohort (*P*=0.898), were not.

Univariate survival analysis of PFS suggested that aberrant p53 staining was associated with a shorter PFS (Dutch cohort: *P*=0.001, HR=1.93, 95% CI: 1.32–2.82; Scottish cohort: *P*=0.038, HR=1.32, 95% CI: 1.02–1.72). A similar trend of p53 on OS was observed (Dutch cohort: *P*=0.084, HR=1.41, 95% CI: 0.96–2.07; Scottish cohort: *P*=0.036, HR=1.35, 95% CI: 1.02–1.80). p53 was not associated with response to chemotherapy in either cohort (Dutch cohort: *P*=0.974; Scottish cohort: *P*=0.139). As the two cohorts were not equally balanced in terms of their clinical characteristics and these may influence the effect of p53, multivariate analysis accounting for all potential confounding factors was essential for further analysis.

The results of multivariate analysis are shown in Table 3. In multivariate analysis for PFS, including country of origin, aberrant p53 staining was not a significant prognostic factor for poor PFS. Country of origin was an independent predictor of PFS; patients in the Scottish cohort tended to have shorter PFS, suggesting that factors other than those measured in this study can influence when a patient progresses (Table 3). Larger residual disease, late stage, higher grade and ‘other’ chemotherapy were also predictors of poor PFS.

For OS, similarly, excessive p53 staining was not associated with poor survival. Larger residual disease, later stage, higher grade and ‘other’ chemotherapy compared to platinum alone were independent predictors of poor OS. This analysis also suggested that patients receiving platinum/taxane combination therapy had better survival rates than patients receiving platinum therapy alone.

No interaction between country of origin and p53 staining was observed to affect outcome (PFS, *P*=0.099; OS, *P*=0.411), suggesting that there were no methodological inconsistencies in the IHC between cohorts that were influencing the survival analysis. Also, no interaction between p53 and chemotherapy was observed to affect outcome (PFS, *P*=0.477; OS, *P*=0.932), suggesting that p53 was not a strong predictive marker of response to chemotherapy in patients in the presence of taxane *vs* non-taxane regimens. Multivariate analysis for factors affecting response to chemotherapy suggested that low-grade (*P*=0.015, OR=0.152, CI: 0.034–0.689) tumours had better response to chemotherapy.

### Multivariate analysis for PFS and OS using the classification proposed by Lassus *et al*

A second classification of p53 IHC staining that groups cases with no p53 staining as aberrant as well as cases with over 50% of cells with moderate or strong intensity staining has been suggested to be prognostic in serous ovarian tumours (Lassus *et al*, 2003). However, independently testing this classification in serous tumours from the present study in the multivariate setting revealed no strong association of p53 with clinical outcome, when account is taken that two classifications of p53 were investigated in the statistical analysis (PFS, *P*=0.094; HR=1.48; OS, *P*=0.035; HR=1.70, *N*=225), whereas residual disease, grade and chemotherapy remained strong (*P*<=0.001) independent prognostic factors in both analyses. Using the response end point, again, p53 had no independent prognostic ability (*P*=0.186; OR=2.98) whereas low grade (*P*=0.020) and the Dutch cohort (*P*=0.037) were significantly associated with better response.

## DISCUSSION

In the past two decades, a wealth of studies has been performed on the prognostic value of p53 expression in ovarian cancer. A recent meta-analysis by Crijns *et al* (2003) on prognostic factors in ovarian cancer demonstrated p53 protein overexpression in 14–79% of ovarian carcinomas. In the same report, data from different studies were pooled, which revealed that patients with aberrant p53 expression had significantly poorer survival at 1 and 5 years. However, owing to the considerable methodological variability among prognostic factor studies, results could only be combined by accepting rather flexible inclusion criteria (Crijns *et al*, 2003).

For the present study, we aimed to analyse the prognostic and predictive impact of p53 expression in a large study population with sufficient statistical power. Our study highlights the importance of standardisation of the methods used for storage and staining of tumour tissue as well as the patient population, data collection and determination of clinical end points. The apparent differing association of p53 staining with classical clinicopathological prognostic factors in the two cohorts could be attributed to differences in the proportions of high- and low-grade patients in the two cohorts. This demonstrates that the particular case mix in a cohort can influence the apparent effect of p53 staining.

Although we minimised variability in the quality of the clinical data by using well-defined patient populations, differences in the clinical characteristics of the patient cohorts meant that multivariate analysis of the prognostic value of p53 was required to account for potentially confounding factors. However, differences in survival between the two cohorts may have also arisen by inconsistent definitions of survival end points, the aggressiveness of chemotherapy or surgery in the two counties, or could have been acting as a surrogate for effects that were not quantified in the analysis such as surgical approach, performance status or deprivation. A recent study has investigated the effect of surgery on clinical outcome of ovarian cancer patients within the context of a clinical trial (Crawford *et al*, 2005). This study indicated that surgical practise differed between the UK and other countries, mainly that more extensive surgery was performed in non-UK countries. This observation may in part explain the differences in PFS between countries, but also suggests that information regarding surgery should be collected and accounted for in future prognostic factor studies.

Methodological variability between the two groups was minimised by performing TMA construction and IHC staining in the same laboratory and by evaluation of all stainings by the same observers. Results of several studies indicate that depending on the fixative used for processing paraffin-embedded tumour tissue, and the storage time of tissue sections, results of IHC staining may vary and these are not routinely mentioned in the literature on ovarian cancer (Prioleau and Schnitt, 1995; Dressler *et al*, 1999; Atkins *et al*, 2004). In breast cancer, standard guidelines for utilisation of formalin-fixed, paraffin-embedded tissue sections have recently been proposed (Dressler *et al*, 1999). Implementation of such guidelines should aid in achieving comparable results among prognostic factor studies. Definitive, reliable evidence for the possible prognostic value of p53 expression should be obtained from large clinical trials with a standardised laboratory protocol and data collection.

Strongly positive p53 staining is mostly associated with missense mutations of the p53 gene. However, the use of IHC staining for determination of p53 status may yield false-positive as well as false-negative results. Positive staining in the absence of p53 mutations may occur when wild-type p53 is activated in response to oncogenic stresses or interaction with viral oncoproteins (Lu *et al*, 1992; Demers *et al*, 1994). Furthermore, stabilisation and accumulation of wild-type p53 may result from disruption of the p53–Mdm2 interaction or the expression of p14ARF (Midgley and Lane, 1997; Zhang *et al*, 1998; Bartel *et al*, 2002). Conversely, false-negative staining may occur in case of homozygous deletion of the p53 gene or by null mutations. Shahin *et al* (2000) performed immunohistochemistry and p53 sequencing on tumour samples of 171 ovarian cancer patients. Their results showed that 32.6% of tumours with a p53 mutation were DO-7 negative, of which 75% carried a null mutation. Patients with p53 null mutations in their tumours had an even poorer survival than patients with missense mutations (Shahin *et al*, 2000). Two recent studies in early and advanced ovarian cancer confirmed that cases with non-missense mutations of the *p53* gene indeed show a low rate of p53 protein accumulation, and that positive p53 immunostaining frequently occurs in tumours with a wild-type *p53* gene. As a result, the concordance between p53 mutation and positive immunostaining was only about 70% (Wang *et al*, 2004a, 2004b).

To avoid false-positive and false-negative staining results, several approaches have been suggested. One approach was suggested by Lassus *et al* (2003), who evaluated the prognostic significance of p53 immunostaining in 522 serous ovarian carcinomas using the TMA technique. Patients were divided into two distinct groups based on DO-7 immunostaining, one with aberrant (negative or strongly positive) p53 expression and a poor disease outcome, and one with normal p53 expression and relatively good outcome. The association of aberrant p53 staining with a poor prognosis was independent of other prognostic factors (Lassus *et al*, 2003). In the present study, we used the same antibody and attempted to independently validate their findings. However, we were not able to confirm their findings in our analysis.

Other approaches that have been used include determination of p53 status by SSCP, direct sequencing or the use of yeast p53 functional assays (FASAY). These approaches, however, are limited by complexity, cost, and collection and storage requirements. Furthermore, mutation does not necessarily correlate with loss of transcriptional activity. Recently, Nenutil *et al* (2005) suggested the combined staining of p53 and mdm2 as a simple and cost-effective method to increase the sensitivity and specificity of p53 determination by IHC staining. Results of their study showed that the combined immunostaining of p53 and mdm2 correctly identifies 86.6% of p53 genotypes, as judged by FASAY.

In order to efficiently study p53 expression in a large cohort and save material from the pathology archives, we have used the TMA technique. This technique was developed by Kononen *et al* (1998) in response to the need for faster approaches to validation of tumour markers. The TMA technique has been validated for different tumour types (Camp *et al*, 2000; Gillett *et al*, 2000; Rosen *et al*, 2004). Rosen *et al* (2004) validated p53 staining on ovarian cancer TMAs and showed that p53 expression of tissue cores correctly represents the expression in a whole slide. The chance of correctly representing a whole section with one 0.6 mm core was 91%. The concordance rate increased to 97% when two cores were evaluable and to 98% when three cores could be evaluated (Rosen *et al*, 2004). To ensure p53 staining in the TMA adequately represented p53 staining in the whole tumour, only cases with two or more assessable cores were included in the analysis for the present study.

Several lines of experimental laboratory-based evidence support the concept that p53 is involved in the cellular response to cytotoxic agents and that loss of p53 is associated with resistance to agents such as cisplatin (Righetti *et al*, 1996; Buttitta *et al*, 1997; Reles *et al*, 2001; Siddik, 2003). In contrast, p53-deficient cell cultures show increased sensitivity to paclitaxel treatment or no difference. Paclitaxel does not directly interact with DNA, but exerts its antitumour activity by stabilising microtubule formation, resulting in cell cycle arrest in the G2–M phase transition. A delayed G1 arrest after paclitaxel treatment could reduce the number of cells with wild-type p53 reaching G2, where paclitaxel exerts its effects (Vasey *et al*, 1996; Wahl *et al*, 1996). Lavarino *et al* (2000), who determined the p53 status of 48 ovarian tumours using SSCP and sequence analysis as well as immunohistochemistry, reported that patients with p53 mutant tumours had an increased sensitivity to paclitaxel in combination with platinum compounds. In the present study, there was no relationship between p53 expression and response to chemotherapy. This is in contrast to previous much smaller studies (Righetti *et al*, 1996; Buttitta *et al*, 1997; Lavarino *et al*, 2000; Reles *et al*, 2001). Furthermore, we have performed logistic regression analysis for the platinum/taxane- and the platinum-treated group separately. P53 was not an independent prognostic factor in these analyses.

In summary, we demonstrated that even with minimal methodological variability, it was inappropriate to combine results from two large, well-defined study populations without appropriately accounting for potential confounding clinical factors. Although strongly positive p53 immunostaining tends to be associated with a poor prognosis in a univariate analysis, this relationship did not hold when accounting for other potentially confounding factors. Standardisation of methods used to store paraffin-embedded tumour tissue and perform IHC analysis, the use of tumour tissue obtained in clinical trials with clearly defined end points and clearly defined, stringent, inclusion criteria, may further elucidate the prognostic impact of p53 immunostaining in the future.

## Figures and Tables

**Table 1:** Clinicopathological characteristics and results of p53 immunostaining

	**UK patients (*n*=288)**	**Dutch patients (*n*=188)**	**All patients (*n*=476)**
*Age (years)*			
Median	58	59	59
Range	23–87	22–83	22–87
			
*PFS (months)*			
Median	13	18	15
Range	0–135	0–158	0–158
			
*OS (months)*			
Median	30	33	31
Range	0–136	37–186	0–186
			
*FIGO stage*			
Stage I	21 (7.3%)	23 (12.1%)	44 (9.2%)
Stage II	39 (13.5%)	18 (9.6%)	57 (12.0%)
Stage III	181 (62.8%)	117 (62.2%)	298 (62.6%)
Stage IV	47 (16.3%)	29 (15.4%)	76 (16.0%)
Missing	0	1 (0.5%)	1 (0.2%)
			
*Tumour type*			
Serous	154 (53.5%)	105 (55.9%)	259 (54.4%)
Mucinous	14 (4.9%)	15 (8.0%)	29 (6.1%)
Clear cell	15 (5.2%)	13 (6.9%)	28 (5.9%)
Endometrioid	36 (12.5%)	26 (13.8%)	62 (13.0%)
Adenocarcinoma	37 (12.8%)	20 (10.6%)	57 (12.0%)
Other	30 (10.4%)	9 (4.8%)	39 (8.2%)
Missing	2 (0.7%)	0	2 (0.4%)
			
*Tumour grade*			
Grade I	19 (6.6%)	23 (12.2%)	47 (9.9%)
Grade II	73 (25.3%)	42 (22.3%)	120 (25.2%)
Grade III	158 (54.9%)	96 (51.1%)	256 (53.8%)
Missing	38 (13.2%)	27 (14.4%)	53 (11.1%)
			
*Residual disease*			
<2 cm	140 (48.6%)	65 (34.6%)	207 (43.5%)
⩾2 cm	142 (49.3%)	110 (58.5%)	250 (52.5%)
Missing	6 (2.1%)	13 (6.9%)	19 (4.0%)
			
*Type of chemotherapy*			
Platinum containing	98 (34.0%)	95 (50.5%)	195 (41.0%)
Platinum and taxane containing	165 (57.3%)	72 (38.3%)	237 (49.8%)
Other regimen	25 (8.7%)	21 (11.2%)	44 (9.2%)
			
*P53 expression*			
Normal	133 (46.2%)	99 (52.7%)	228 (47.9%)
Aberrant	155 (53.8%)	89 (47.3%)	248 (52.1%)

FIGO=International Federation of Gynaecology and Obstetrics; OS=overall survival; PFS=progression-free survival.

**Table 2:** Relationship of p53 expression with clinicopathological characteristics

	**UK patients**			**Dutch patients**		
	**Normal p53**	**Excessive p53**	***P*-value^a^**	**Normal p53**	**Excessive p53**	***P*-value^a^**
*Age (years)*						
<58	71 (52.2%)	65 (47.8%)	0.709	50 (56.2%)	39 (43.8%)	0.383
⩾58	76 (50.0%)	76 (50.0%)		49 (49.5%)	50 (50.5%)	
						
*FIGO stage*						
Stage I/II	30 (50.0%)	30 (50.0%)	0.856	29 (70.7%)	12 (29.3%)	**0.006**
Stage III/IV	117 (51.3%)	111 (48.7%)		69 (47.3%)	77 (52.7%)	
						
*Tumour type*						
Serous	74 (48.1%)	80 (51.9%)	0.273	48 (45.7%)	57 (54.3%)	**0.040**
Non-serous	72 (54.5%)	68 (45.5%)		51 (61.4%)	32 (38.6%)	
						
*Differentiation grade*						
Grade I	16 (84.2%)	3 (15.8%)	**0.003**	26 (92.9%)	2 (7.1%)	**<0.001**
Grade II/III	112 (48.5%)	119 (51.5%)		64 (44.1%)	81 (55.9%)	
						
*Residual disease*						
<2 cm	74 (52.9%)	66 (47.1%)	0.404	44 (67.7%)	21 (32.3%)	**0.002**
⩾2 cm	68 (47.9%)	74 (52.1%)		49 (44.5%)	61 (55.5%)	
						
*Response to chemotherapy*						
CR/PR	27 (41.5%)	58 (58.5%)	0.139	39 (70.9%)	42 (71.2%)	0.974
SD/PD	21 (56.8%)	16 (43.2%)		16 (29.1%)	17 (28.8%)	

CR=complete response; FIGO=International Federation of Gynaecology and Obstetrics; PD=progressive disease; PR=partial response; SD=stable disease.

a *P*-values were calculated using *χ*^2^ or Fisher's exact test, where appropriate.

Bold signifies *P*<0.05.

**Table 3:** Multivariate analysis of p53 (50%) on PFS and OS in Dutch and Scottish patients (cohorts combined)

	**PFS**			**OS**		
	***P*-value**	**HR**	**95% CI**	***P*-value**	**HR**	**95% CI**
Dutch cohort^a^	0.036	0.76	0.59–0.98	0.101	0.80	0.61–1.05
Age>58 years	0.31	1.13	0.89–1.44	0.072	1.27	0.98–1.63
Residual disease >2 cm	<0.001	1.97	1.52–2.57	<0.001	1.94	1.47–2.57
Non-serous tumour type	0.092	0.81	0.64–1.04	0.611	0.94	0.72–1.21
Stage III/IV	<0.001	2.14	1.45–3.17	<0.001	2.12	1.38–3.25
Grade II/III	0.001	2.53	1.45–4.44	0.001	2.65	1.46–4.79
Chemotherapy	<0.001			<0.001		
Platinum *vs* taxane and platinum	0.237	0.86	0.67–1.10	0.004	0.67	0.51–0.88
Other *vs* platinum	<0.001	2.86	1.88–4.37	<0.001	2.46	1.61–3.73
Aberrant p53 staining	0.228	1.16	0.91–1.47	0.362	1.13	0.87–1.45

CI=confidence interval; HR=hazard ratio; OS=overall survival; PFS=progression-free survival.

a Categories are given relative to the baseline comparator group.
